# Data on the expression of cellular lncRNAs in human adenovirus infected cells

**DOI:** 10.1016/j.dib.2016.06.053

**Published:** 2016-07-05

**Authors:** Maoshan Chen, Hongxing Zhao, Sara Bergström Lind, Ulf Pettersson

**Affiliations:** a Department of Biochemistry and Genetics, La Trobe Institute for Molecular Science, La Trobe University, Melbourne, Victoria 3086, Australia; b Department of Chemistry-BMC, Analytical Chemistry, Science for Life Laboratory, Uppsala University, Box 599, SE-751 24 Uppsala, Sweden; c The Beijer Laboratory, Department of Immunology, Genetics and Pathology, Uppsala University, S-751 85 Uppsala, Sweden

**Keywords:** Adenovirus, Long noncoding RNA, IncRNA, IMR-90

## Abstract

Expression of cellular long non-coding RNAs (lncRNAs) in human primary lung fibroblasts (IMR-90) during the course of adenovirus type 2 (Ad2) infection was studied by strand-specific whole transcriptome sequencing. In total, 645 cellular lncRNAs were expressed at a significant level and 398 of them were changed more than 2-fold. The changes in expression followed a distinct temporal pattern. Significantly, 80% of the changes occurred at the late phase and 80% of the de-regulated lncRNAs were up-regulated. The three largest groups of deregulated lncRNAs were 125 antisense RNAs, 111 pseudogenes and 85 long intergenic non-coding RNAs (lincRNAs). Lastly, more than 36% of lncRNAs have been shown to interact with RNA binding proteins.

## **Specifications Table**

**Table:** 

Subject area	Biology
More specific subject area	Gene expression
Type of data	Tables
How data was acquired	LncRNA expression was measured by paired-end cDNA sequencing using an Illumina HiSeq 2000 sequencer.
Data format	Filtered, processed
Experimental factors	Human primary lung fibroblast cells were infected with Ad2 and RNA was extracted after 6, 12, 24, and 36 h. Uninfected cells were used as control.
Experimental features	Differentially expressed lncRNAs required that their expression level was more than 10 FPKM (Fragments per Kilobase of exon per Million fragments mapped) and that the minimal change was 2-fold
Data source location	Uppsala university, Sweden
Data accessibility	Data is presented within this article

## **Value of the data**

• Provide unique insights into the changes in lncRNA expression in human primary lung fibroblasts during an adenovirus infection.
• Provide a valuable and unique resource for studies of lncRNAs expression and regulation.
• Provide unique insights in the regulation of cellular gene expression mediated by lncRNAs.
• Provide clues to our understanding of lncRNA biological function.
• Since the effect of adenovirus on host cells in the early phase mimics tumorigenesis by promoting cell growth and inhibiting apoptosis, our data are applicable to cancer research.

## 1 Data

Using pair-end sequencing, 398 cellular lncRNAs were identified as differentially expressed more than 2-fold in IMR-90 cells during the course of Ad2 infection. According to GENCODE, 125 are antisense RNAs, 111 are pseudogenes and 85 are long intergenic non-coding RNAs (lincRNA). Based on their expression profiles, these lncRNAs fell into 10 major clusters. The list of differentially expressed lncRNAs, sequencing reads, fold change, biotypes, expression cluster as well as their lengths and location on the genome are included in Table S1. Among differentially expressed lncRNAs, 149 lncRNAs have been shown to interact with RNA binding proteins (RBPs) (Table 1). In total, 33 RBPs proteins have been proved to interact with these lncRNAs. Furthermore, we showed here that 21 and 15 out of 33 RBPs are detected at mRNA and protein level, respectively (Table 2**)**.

## 2 Experimental design, materials and methods

### 2.1 Cell culture and Ad2 infection

Human primary lung fibroblast cells (IMR-90) were cultured in a complete Eagle׳s minimum essential medium (10% fetal bovine serum, 100 U/ml penicillin and 100 µg/ml streptomycin). After reaching confluence, the cells were cultured for two more days to reach growth synchronization [1]. Cells were mock-infected or infected with Ad2 at a multiplicity of infection (MOI) of 100 fluorescence-forming units (FFU) per cell in serum-free medium. After 1 h adsorption at 37 °C, the medium was replaced with complete medium and incubated at 37 °C. Infected cells were collected at 6, 12, 24, and 36 hours post-infection (hpi). Mock-infected cells were collected at 6 hpi.

### 2.2 RNA extraction, cDNA library preparation, and sequencing

Total RNAs were extracted using TRIZOL Reagent (Invitrogen). The quality of the input RNA was controlled by the Agilent 2100 Bioanalyzer (Agilent Technologies). Purified RNAs were treated with RiboZero (Epicenter) to remove ribosomal RNAs and cDNA libraries were constructed using ScriptSeq™ v2 RNA-Seq library preparation kit according to the manufacturer׳s protocol (Epicenter). The cDNA libraries were sequenced using Illumina HiSeq 2000.

### 2.3 Bioinformatics analysis

After data cleaning, the reads were aligned to human genome sequences (GRCh38, Ensembl) with TopHat2 software [2]. TopHat2 incorporates Bowtie2 (http://bowtie-bio.sourceforge.net/bowtie2/index.shtml) algorithm to perform the alignment. We used default parameters which allowed a maximum of two mismatches when mapping the reads to the human genome. Cufflinks was then used to profile gene expression at each time point based on human gene annotation by Ensembl [3]. Differentially expressed lncRNAs were identified by three statistical values. 1), fold change was calculated by the FPKM (Fragments per Kilobase of exon per Million fragments mapped) values between Ad2-infected to uninfected cells; 2), based on Poison distribution, *p*-values were used to present the significances of differentially expressed lncRNAs [4]; 3), using the NOIseq package, the probability of a differentially expressed lncRNA was calculated [5]. The hierarchical lncRNAs with different expression patterns were analyzed with uncentered correlation and centroid linkage method by Cluster and Tree View software.

### 2.4 Expression of lncRNA binding proteins

All the proteins that interacted with lncRNAs were downloaded from starBase v2.0 which is based on CHIP-Seq analysis (http://starbase.sysu.edu.cn) [6]. mRNA expression data was extracted from the current data. Whereas the protein expression data was obtained by SILAC-MS using the same cell culture and infection condition (manuscript in preparation). Briefly, IMR-90 cells were cultured in cell culture medium for stable isotope labeling by amino acids in cell culture (SILAC) for at least six population doublings. Cells labeled with heavy or light amino acids were then infected with Ad2 or mock infected, respectively. A biological replicate with swapped labeling was also performed. Mock- and Ad2-infected lysates of different labeling were combined in a 1:1 protein ratio. Proteins were fractionated using SDS-PAGE and each lane was cut into ten pieces. Following in-gel tryptic digestion, peptides were extracted and analyzed using QExactive Orbitrap Plus Mass spectrometer (ThermoFisher Scientific, Bremen, Germany) Acquired data (raw-files) was imported into MaxQuant software (version: 1.4) and searched against a FASTA-file containing both cellular and Ad2 proteins. The ratio of the chromatographic areas of heavy and light peptides matching to specific proteins was used for determining the differences in protein expression. The reported values are the average of two biological replicates.

## Figures and Tables

**Table 1:** LncRNAs interacting with RNA binding proteins.

**Tracking_id**	**Locus**	**Length**	**lncRNA**	**Biotype**	**No. of Interacted RBP**	**RBP**
ENSG00000188206	1:244840637-244846903	6267	HNRNPU-AS1	antisense	31	HuR,PTB,IGF2BP1,IGF2BP2,IGF2BP3,PUM2,QKI,TNRC6,eIF4AIII,DGCR8,FMRP,FXR1,FXR2,FUS,LIN28A,LIN28B,LIN28,MOV10,ALKBH5,C22ORF28,CAPRIN1,ZC3H7B,EWSR1,FUS-mutant,TAF15,SFRS1,U2AF65,TIA1,TIAL1,hnRNPC,UPF1,
ENSG00000255717	11:62851987-62855914	3928	SNHG1	processed_transcript	31	TIAL1,hnRNPC,DGCR8,SFRS1,TIA1,ALKBH5,HuR,PTB,IGF2BP3,EWSR1,TDP43,FUS,ZC3H7B,FUS-mutant,U2AF65,QKI,MOV10,CAPRIN1,IGF2BP1,IGF2BP2,TNRC6,eIF4AIII,LIN28B,C22ORF28,UPF1,FMRP,FXR2,C17ORF85,TAF15,LIN28A,LIN28,
ENSG00000260032	20:36045621-36050960	5340	LINC00657	lincRNA	31	HuR,PTB,IGF2BP1,IGF2BP2,IGF2BP3,PUM2,QKI,TNRC6,eIF4AIII,DGCR8,FMRP,FXR1,FXR2,FUS,LIN28A,LIN28B,MOV10,C17ORF85,C22ORF28,CAPRIN1,ZC3H7B,EWSR1,FUS-mutant,TAF15,SFRS1,U2AF65,TIA1,TIAL1,hnRNPC,UPF1,TDP43,
ENSG00000229807	X:73820650-73852753	32,104	XIST	lincRNA	29	TNRC6,eIF4AIII,DGCR8,C22ORF28,TIA1,FMRP,FXR1,LIN28A,LIN28,C17ORF85,CAPRIN1,ZC3H7B,IGF2BP1,IGF2BP2,IGF2BP3,PUM2,FXR2,MOV10,ALKBH5,EWSR1,SFRS1,FUS,LIN28B,FUS-mutant,TIAL1,UPF1,TDP43,HuR,TAF15,
ENSG00000247556	15:41283989-41309737	25,749	OIP5-AS1	processed_transcript	29	HuR,PTB,IGF2BP1,IGF2BP2,IGF2BP3,PUM2,TNRC6,eIF4AIII,DGCR8,FMRP,FXR1,FXR2,FUS,LIN28A,LIN28B,LIN28,MOV10,CAPRIN1,ZC3H7B,EWSR1,FUS-mutant,TAF15,SFRS1,U2AF65,TIA1,TIAL1,hnRNPC,UPF1,TDP43,
ENSG00000163597	17:76557765-76565348	7584	SNHG16	processed_transcript	27	PTB,IGF2BP1,IGF2BP2,IGF2BP3,QKI,eIF4AIII,DGCR8,FMRP,FXR2,FUS,LIN28A,LIN28B,LIN28,MOV10,C17ORF85,C22ORF28,ZC3H7B,EWSR1,FUS-mutant,TAF15,SFRS1,U2AF65,TIA1,TIAL1,hnRNPC,UPF1,TDP43,
ENSG00000203875	6:85660949-85678736	17,788	SNHG5	processed_transcript	27	hnRNPC,TNRC6,TIAL1,UPF1,QKI,FMRP,ZC3H7B,PUM2,eIF4AIII,C17ORF85,EWSR1,TDP43,PTB,FXR2,FUS,SFRS1,DGCR8,CAPRIN1,HuR,IGF2BP1,IGF2BP2,C22ORF28,U2AF65,IGF2BP3,LIN28A,LIN28,LIN28B,
ENSG00000242125	1:28505979-28510892	4914	SNHG3	sense_intronic	25	HuR,PTB,IGF2BP1,IGF2BP2,IGF2BP3,eIF4AIII,DGCR8,FMRP,FXR2,FUS,LIN28A,LIN28B,LIN28,C22ORF28,ZC3H7B,EWSR1,FUS-mutant,TAF15,SFRS1,U2AF65,TIA1,TIAL1,hnRNPC,UPF1,TDP43,
ENSG00000245532	11:65422773-65445540	22,768	NEAT1	lincRNA	25	TNRC6,C22ORF28,eIF4AIII,TDP43,FMRP,TAF15,TIA1,DGCR8,FUS,ZC3H7B,FUS-mutant,UPF1,IGF2BP3,QKI,ALKBH5,EWSR1,hnRNPC,PTB,IGF2BP1,SFRS1,IGF2BP2,TIAL1,U2AF65,LIN28A,LIN28B,
ENSG00000245910	8:66921683-66926398	4716	SNHG6	processed_transcript	24	hnRNPC,C17ORF85,ZC3H7B,HuR,TNRC6,FUS,TIAL1,TDP43,PUM2,MOV10,U2AF65,TIA1,UPF1,IGF2BP2,IGF2BP3,eIF4AIII,CAPRIN1,IGF2BP1,LIN28B,DGCR8,LIN28A,LIN28,FMRP,C22ORF28,
ENSG00000245694	16:54918862-54929189	10,328	CRNDE	lincRNA	23	HuR,PTB,IGF2BP1,IGF2BP2,IGF2BP3,TNRC6,eIF4AIII,DGCR8,FMRP,FXR2,FUS,LIN28A,LIN28B,LIN28,ZC3H7B,EWSR1,FUS-mutant,TAF15,U2AF65,TIA1,TIAL1,hnRNPC,UPF1,
ENSG00000233016	9:136721365-136728184	6820	SNHG7	antisense	22	hnRNPC,IGF2BP3,IGF2BP2,ZC3H7B,FMRP,EWSR1,U2AF65,HuR,DGCR8,FUS,C22ORF28,FUS-mutant,SFRS1,UPF1,TDP43,IGF2BP1,eIF4AIII,TAF15,ALKBH5,LIN28B,LIN28A,LIN28,
ENSG00000259001	14:20343047-20343685	639	RPPH1	antisense	20	HuR,PTB,TNRC6,eIF4AIII,DGCR8,FMRP,FXR2,FUS,LIN28A,LIN28B,ALKBH5,C17ORF85,C22ORF28,CAPRIN1,ZC3H7B,TAF15,U2AF65,TIAL1,UPF1,TDP43,
ENSG00000232956	7:44983022-44986961	3940	SNHG15	lincRNA	20	HuR,eIF4AIII,DGCR8,FMRP,FXR2,FUS,LIN28A,LIN28,C22ORF28,CAPRIN1,ZC3H7B,EWSR1,TAF15,SFRS1,U2AF65,TIA1,TIAL1,hnRNPC,UPF1,TDP43,
ENSG00000231607	13:49982551-50125720	143,170	DLEU2	antisense	19	TIAL1,hnRNPC,IGF2BP3,FUS,FUS-mutant,U2AF65,TIA1,IGF2BP1,FMRP,ZC3H7B,EWSR1,HuR,IGF2BP2,eIF4AIII,DGCR8,LIN28B,PUM2,UPF1,TAF15,
ENSG00000269893	4:118278708-118279823	1116	SNHG8	lincRNA	19	HuR,eIF4AIII,DGCR8,FMRP,FUS,LIN28A,LIN28,MOV10,C22ORF28,CAPRIN1,ZC3H7B,EWSR1,TAF15,SFRS1,U2AF65,TIA1,hnRNPC,UPF1,TDP43,
ENSG00000258441	14:21200078-21206900	6823	LINC00641	processed_transcript	19	PTB,IGF2BP1,IGF2BP2,IGF2BP3,eIF4AIII,DGCR8,FMRP,FXR2,FUS,LIN28B,CAPRIN1,ZC3H7B,EWSR1,FUS-mutant,U2AF65,TIAL1,hnRNPC,UPF1,TDP43,
ENSG00000243960	1:111438637-111441364	2728	RP11-552M11.4	sense_overlapping	19	HuR,PTB,IGF2BP1,IGF2BP2,IGF2BP3,eIF4AIII,FMRP,FUS,LIN28A,LIN28B,LIN28,ZC3H7B,FUS-mutant,U2AF65,TIA1,TIAL1,hnRNPC,UPF1,TDP43,
ENSG00000215417	13:91347819-91354579	6761	MIR17HG	processed_transcript	18	SFRS1,TIA1,TIAL1,DGCR8,EWSR1,FUS,HuR,MOV10,PTB,FMRP,LIN28A,LIN28B,hnRNPC,UPF1,FXR1,U2AF65,eIF4AIII,ZC3H7B,
ENSG00000197989	1:28578537-28582983	4447	SNHG12	antisense	18	TDP43,DGCR8,TIAL1,EWSR1,FUS,SFRS1,C22ORF28,U2AF65,FMRP,C17ORF85,HuR,eIF4AIII,FXR2,UPF1,LIN28A,LIN28,hnRNPC,LIN28B,
ENSG00000226950	4:52712403-52720351	7949	DANCR	processed_transcript	18	HuR,PTB,IGF2BP1,IGF2BP2,IGF2BP3,eIF4AIII,DGCR8,FMRP,FUS,LIN28B,LIN28,CAPRIN1,FUS-mutant,TAF15,U2AF65,TIA1,TIAL1,UPF1,
ENSG00000177410	20:49278177-49295738	17,562	ZFAS1	antisense	18	HuR,IGF2BP1,IGF2BP2,IGF2BP3,eIF4AIII,FMRP,FUS,LIN28A,LIN28,MOV10,C22ORF28,CAPRIN1,ZC3H7B,FUS-mutant,TAF15,SFRS1,U2AF65,UPF1,
ENSG00000276232	12:6510274-6510522	249	SCARNA10	sense_intronic	18	PTB,eIF4AIII,DGCR8,FMRP,FXR2,FUS,LIN28A,LIN28B,LIN28,C17ORF85,C22ORF28,ZC3H7B,EWSR1,U2AF65,TIAL1,hnRNPC,UPF1,TDP43,
ENSG00000170846	4:6673450-6676047	2598	AC093323.3	lincRNA	17	HuR,PTB,IGF2BP1,IGF2BP2,IGF2BP3,eIF4AIII,DGCR8,FMRP,FUS,MOV10,CAPRIN1,ZC3H7B,U2AF65,TIA1,TIAL1,hnRNPC,UPF1,
ENSG00000179818	2:69962262-70088846	126,585	PCBP1-AS1	antisense	16	PTB,eIF4AIII,DGCR8,FMRP,FXR2,FUS,LIN28A,LIN28B,LIN28,SFRS1,U2AF65,TIA1,TIAL1,hnRNPC,UPF1,TDP43,
ENSG00000267575	19:27793462-27918863	125,402	CTC-459F4.3	processed_transcript	16	PTB,IGF2BP1,IGF2BP2,IGF2BP3,QKI,eIF4AIII,DGCR8,FMRP,FUS,LIN28A,C22ORF28,TAF15,U2AF65,TIAL1,hnRNPC,UPF1,
ENSG00000270066	1:109100192-109100619	428	SCARNA2	lincRNA	15	PTB,eIF4AIII,DGCR8,FMRP,FXR2,FUS,LIN28A,LIN28B,MOV10,C17ORF85,U2AF65,TIAL1,hnRNPC,UPF1,TDP43,
ENSG00000251022	4:82893008-82900960	7953	THAP9-AS1	antisense	15	IGF2BP1,PUM2,eIF4AIII,DGCR8,FUS,LIN28B,LIN28,CAPRIN1,FUS-mutant,TAF15,U2AF65,TIA1,TIAL1,hnRNPC,UPF1,
ENSG00000261553	13:75549772-75807120	257,349	RP11-29G8.3	processed_transcript	15	HuR,PTB,IGF2BP1,IGF2BP2,TNRC6,eIF4AIII,DGCR8,FMRP,FXR2,FUS,C22ORF28,SFRS1,U2AF65,hnRNPC,UPF1,
ENSG00000254911	11:93721512-93721865	354	SCARNA9	antisense	14	PTB,eIF4AIII,DGCR8,FMRP,FXR2,FUS,C17ORF85,ZC3H7B,EWSR1,FUS-mutant, TIAL1, hnRNPC, UPF1,TDP43,
ENSG00000260035	15:45200324-45200632	309	CTD-2651B20.6	sense_intronic	14	PTB,IGF2BP2,eIF4AIII,DGCR8,FMRP,LIN28A,LIN28B,LIN28,SFRS1,TIA1,TIAL1,hnRNPC,UPF1,TDP43,
ENSG00000230590	X:73963954-74293574	329,621	FTX	lincRNA	14	IGF2BP1,IGF2BP2,IGF2BP3,eIF4AIII,DGCR8,FUS,ZC3H7B,SFRS1,U2AF65,TIA1,TIAL1,hnRNPC,UPF1,TDP43,
ENSG00000126005	20:35216461-35278131	61,671	MMP24-AS1	antisense	13	HuR,IGF2BP1,IGF2BP2,IGF2BP3,eIF4AIII,DGCR8,FMRP,FUS,LIN28A,ZC3H7B,U2AF65,TIA1,UPF1,
ENSG00000267321	17:35568119-35574792	6674	RP11-1094M14.11	lincRNA	13	IGF2BP1,IGF2BP2,IGF2BP3,eIF4AIII,FMRP,FUS,LIN28A,LIN28B,ZC3H7B,FUS-mutant,TAF15,U2AF65,UPF1,
ENSG00000226688	10:95753205-96090238	337,034	ENTPD1-AS1	antisense	12	HuR,PTB,eIF4AIII,FMRP,FUS,LIN28B,EWSR1,U2AF65,TIA1,TIAL1,hnRNPC,UPF1,
ENSG00000264112	17:57989038-57994850	5813	RP11-159D12.2	lincRNA	12	HuR,PTB,IGF2BP1,IGF2BP2,IGF2BP3,eIF4AIII,DGCR8,FUS,ZC3H7B,TAF15,U2AF65,hnRNPC,
ENSG00000233429	7:27095646-27100265	4620	HOTAIRM1	antisense	12	PTB,IGF2BP1,eIF4AIII,DGCR8,FUS,LIN28A,CAPRIN1,SFRS1,U2AF65,TIAL1,hnRNPC,UPF1,
ENSG00000234327	17:5111467-5115004	3538	AC012146.7	processed_transcript	12	IGF2BP1,IGF2BP2,IGF2BP3,eIF4AIII,DGCR8,LIN28B,EWSR1,FUS-mutant,TAF15,SFRS1,U2AF65,TIAL1,
ENSG00000223546	X:102769160-102885406	116,247	LINC00630	lincRNA	11	IGF2BP2,IGF2BP3,eIF4AIII,DGCR8,FMRP,FUS,LIN28B,U2AF65,TIAL1,hnRNPC,UPF1,
ENSG00000186594	17:1711492-1717174	5683	MIR22HG	lincRNA	11	HuR,PTB,eIF4AIII,DGCR8,FMRP,FUS,LIN28A,LIN28B,ZC3H7B,U2AF65,UPF1,
ENSG00000247828	5:88268894-88436685	167,792	TMEM161B-AS1	antisense	10	PTB,IGF2BP3,eIF4AIII,DGCR8,FUS,SFRS1,U2AF65,TIAL1,hnRNPC,UPF1,
ENSG00000247092	14:95532296-95534872	2577	SNHG10	antisense	10	U2AF65,hnRNPC,FUS,LIN28B,eIF4AIII,ZC3H7B,UPF1,HuR,TAF15,FMRP,
ENSG00000142396	19:58305318-58315663	10,346	ERVK3-1	processed_transcript	10	PTB,eIF4AIII,DGCR8,FMRP,FUS,LIN28A,LIN28B,U2AF65,TIAL1,UPF1,
ENSG00000261889	16:3156735-3157483	749	RP11-473M20.16	lincRNA	10	eIF4AIII,DGCR8,FMRP,FXR2,LIN28A,LIN28B,EWSR1,TIA1,TIAL1,UPF1,
ENSG00000196295	7:30516308-30594809	78,502	AC005154.6	processed_transcript	10	PTB,eIF4AIII,DGCR8,FUS,LIN28B,EWSR1,U2AF65,TIAL1,hnRNPC,UPF1,
ENSG00000261061	16:81030769-81031485	717	RP11-303E16.2	sense_intronic	10	HuR,PTB,IGF2BP1,IGF2BP2,IGF2BP3,TNRC6,eIF4AIII,DGCR8,U2AF65,UPF1,
ENSG00000255198	16:1964958-1965509	552	SNHG9	lincRNA	9	CAPRIN1,U2AF65,FUS,HuR,DGCR8,FMRP,UPF1,eIF4AIII,C22ORF28,
ENSG00000231312	2:39436636-39665343	228,708	AC007246.3	antisense	9	PTB,eIF4AIII,DGCR8,FUS,U2AF65,TIA1,TIAL1,UPF1,TDP43,
ENSG00000258297	11:66666035-66668374	2340	RP11-658F2.8	antisense	8	IGF2BP1,IGF2BP2,IGF2BP3,eIF4AIII,FUS,ZC3H7B,U2AF65,UPF1,
ENSG00000234608	12:111839763-111842902	3140	MAPKAPK5-AS1	lincRNA	8	IGF2BP1,IGF2BP3,eIF4AIII,FMRP,FUS,LIN28A,U2AF65,UPF1,
ENSG00000230844	X:46545492-46548408	2917	ZNF674-AS1	lincRNA	8	HuR,PTB,eIF4AIII,FMRP,LIN28B,U2AF65,hnRNPC,UPF1,
ENSG00000258486	14:49586578-49586878	301	RN7SL1	known_ncrna	8	DGCR8,FMRP,FXR2,LIN28A,LIN28B,MOV10,hnRNPC,TDP43,
ENSG00000228549	1:16870944-16874092	3149	RP11-108M9.3	lincRNA	8	PTB,DGCR8,LIN28A,U2AF65,TIA1,TIAL1,hnRNPC,UPF1,
ENSG00000224078	15:24978582-25056565	77,984	SNHG14	processed_transcript	8	PTB,eIF4AIII,DGCR8,FUS,LIN28,TIAL1,UPF1,TDP43,
ENSG00000229152	13:110894638-110899172	4535	ANKRD10-IT1	sense_intronic	7	IGF2BP1,IGF2BP2,IGF2BP3,eIF4AIII,FUS,U2AF65,hnRNPC,
ENSG00000253552	7:27107776-27134302	26,527	HOXA-AS2	antisense	7	eIF4AIII,DGCR8,FMRP,FUS,U2AF65,UPF1,TDP43,
ENSG00000240498	9:21994777-22121097	126,321	CDKN2B-AS1	antisense	7	PTB,eIF4AIII,DGCR8,FUS,LIN28A,U2AF65,UPF1,
ENSG00000261526	19:1874870-1876169	1300	CTB-31O20.2	lincRNA	7	IGF2BP1,eIF4AIII,DGCR8,FMRP,FUS,U2AF65,hnRNPC,
ENSG00000222041	2:87455367-87606805	151,439	LINC00152	lincRNA	7	PTB,eIF4AIII,FUS,U2AF65,TIA1,TIAL1,UPF1,
ENSG00000236088	17:13756477-14069495	313,019	COX10-AS1	processed_transcript	6	PTB,eIF4AIII,U2AF65,TIAL1,hnRNPC,UPF1,
ENSG00000269825	19:52650436-52653284	2849	CTD-3099C6.9	sense_intronic	6	PTB,IGF2BP2,eIF4AIII,FMRP,LIN28B,UPF1,
ENSG00000268388	16:86474528-86509099	34,572	FENDRR	lincRNA	6	PTB,eIF4AIII,DGCR8,FUS,hnRNPC,UPF1,
ENSG00000236824	2:47331059-47344517	13,459	BCYRN1	lincRNA	6	eIF4AIII,FUS,U2AF65,TIAL1,UPF1,TDP43,
ENSG00000255248	11:122028354-122116323	87,970	RP11-166D19.1	sense_overlapping	6	eIF4AIII,DGCR8,FUS,U2AF65,hnRNPC,UPF1,
ENSG00000255090	11:122155421-122422871	267,451	RP11-820L6.1	lincRNA	6	eIF4AIII,U2AF65,TIA1,TIAL1,hnRNPC,UPF1,
ENSG00000233461	1:231522387-231528556	6170	RP11-295G20.2	antisense	5	IGF2BP1,IGF2BP2,IGF2BP3,eIF4AIII,UPF1,
ENSG00000255135	11:76441337-76444656	3320	RP11-111M22.3	lincRNA	5	eIF4AIII,LIN28,U2AF65,TIAL1,UPF1,
ENSG00000261094	9:122937622-122940333	2712	RP11-355O1.11	sense_overlapping	5	PTB,eIF4AIII,U2AF65,hnRNPC,UPF1,
ENSG00000232442	20:63627226-63628824	1599	CTD-3184A7.4	antisense	5	eIF4AIII,DGCR8,FUS,U2AF65,UPF1,
ENSG00000233421	1:16533885-16536172	2288	RP5-875O13.1	lincRNA	5	eIF4AIII,DGCR8,FMRP,LIN28,U2AF65,
ENSG00000214548	14:100779409-100861031	81,623	MEG3	lincRNA	5	PTB,eIF4AIII,DGCR8,FUS,TDP43,
ENSG00000247137	11:83184490-83193794	9305	RP11-727A23.5	processed_transcript	5	eIF4AIII,DGCR8,U2AF65,TIAL1,hnRNPC,
ENSG00000248690	8:121639292-121644693	5402	HAS2-AS1	antisense	4	PTB,eIF4AIII,FUS,UPF1,
ENSG00000237491	1:778769-810060	31,292	RP11-206L10.9	lincRNA	4	eIF4AIII,FUS,SFRS1,U2AF65,
ENSG00000224505	17:45150399-45161510	11,112	AC002117.1	antisense	4	eIF4AIII,FUS,U2AF65,UPF1,
ENSG00000269926	10:72274914-72275980	1067	RP11-442H21.2	antisense	3	eIF4AIII,DGCR8,UPF1,
ENSG00000256813	11:60841805-60851081	9277	RP11-804A23.4	antisense	3	DGCR8,FUS,UPF1,
ENSG00000233101	17:48549629-48606414	56,786	HOXB-AS3	antisense	3	PTB,LIN28A,LIN28B,
ENSG00000269243	19:16123660-16139892	16,233	CTD-2231E14.8	antisense	3	eIF4AIII,FUS,UPF1,
ENSG00000260708	22:46761893-46762563	671	CTA-29F11.1	antisense	3	eIF4AIII,FUS,U2AF65,
ENSG00000271335	10:35314551-35336401	21,851	RP11-324I22.4	antisense	3	eIF4AIII,FUS,UPF1,
ENSG00000261054	15:99128831-99131806	2976	RP11-6O2.4	antisense	3	HuR,eIF4AIII,FUS,
ENSG00000266490	17:30792371-30792833	463	CTD-2349P21.9	lincRNA	3	eIF4AIII,FUS,UPF1,
ENSG00000270195	4:1712820-1713622	803	RP11-572O17.1	lincRNA	3	eIF4AIII,FUS,U2AF65,
ENSG00000219665	19:11987616-12046275	58,660	CTD-2006C1.2	processed_transcript	3	IGF2BP1,IGF2BP2,eIF4AIII,
ENSG00000223343	3:48985048-48989988	4941	RP13-131K19.2	antisense	3	eIF4AIII,FUS,UPF1,
ENSG00000261220	8:133573182-133573861	680	RP11-629O1.2	lincRNA	3	PTB,eIF4AIII,UPF1,
ENSG00000269051	19:53197110-53211015	13,906	CTD-2245F17.3	lincRNA	3	eIF4AIII,FUS,SFRS1,
ENSG00000225511	9:92141297-92160114	18,818	LINC00475	lincRNA	3	PTB,eIF4AIII,FUS,
ENSG00000245573	11:27506837-27698174	191,338	BDNF-AS	antisense	3	eIF4AIII,FUS,UPF1,
ENSG00000224959	2:111491942-111494811	2870	AC017002.2	lincRNA	3	eIF4AIII,FUS,TIAL1,
ENSG00000249669	5:149406688-149428678	21,991	MIR143HG	lincRNA	3	FUS,TIAL1,hnRNPC,
ENSG00000230606	2:97416164-97433527	17,364	AC159540.1	lincRNA	2	eIF4AIII,FUS,
ENSG00000258399	14:100894769-100935999	41,231	MEG8	lincRNA	2	TDP43,FUS,
ENSG00000256940	11:64245963-64248217	2255	RP11-783K16.5	antisense	2	eIF4AIII,UPF1,
ENSG00000269604	19:4791744-4795559	3816	AC005523.2	antisense	2	FUS,UPF1,
ENSG00000267776	19:56376703-56377284	582	AC006116.24	sense_intronic	2	FUS,EWSR1,
ENSG00000267458	19:12944117-12944487	371	CTC-425F1.4	antisense	2	FUS,UPF1,
ENSG00000257553	12:56104613-56113905	9293	RP11-603J24.17	antisense	2	eIF4AIII,UPF1,
ENSG00000238045	16:29808635-29821252	12,618	AC009133.12	antisense	2	eIF4AIII,U2AF65,
ENSG00000259952	16:29806495-29807732	1238	AC009133.15	antisense	2	eIF4AIII,LIN28B,
ENSG00000228109	3:196999459-197004744	5286	MFI2-AS1	antisense	2	eIF4AIII,U2AF65,
ENSG00000261822	15:42567030-42569994	2965	RP11-265N6.2	antisense	2	eIF4AIII,UPF1,
ENSG00000260934	16:19501688-19502286	599	CTA-363E6.7	antisense	2	eIF4AIII,FUS,
ENSG00000240801	11:2129120-2129964	845	AC132217.4	3prime_overlapping_ncrna	2	eIF4AIII,FUS,
ENSG00000227896	10:86521944-86525101	3158	RP11-77P6.2	antisense	2	eIF4AIII,FUS,
ENSG00000254452	11:66276778-66277492	715	RP11-867G23.4	antisense	2	FUS,UPF1,
ENSG00000259357	1:150965244-150966256	1013	RP11-316M1.12	antisense	2	eIF4AIII,UPF1,
ENSG00000269968	12:6537793-6538370	578	RP5-940J5.9	antisense	2	eIF4AIII,UPF1,
ENSG00000260923	16:90185996-90222678	36,683	AC137934.1	lincRNA	2	eIF4AIII,FUS,
ENSG00000269439	19:17488989-17511889	22,901	CTD-3131K8.2	lincRNA	2	eIF4AIII,FUS,
ENSG00000261602	16:69709873-69710583	711	CTD-2033A16.1	antisense	2	eIF4AIII,UPF1,
ENSG00000234961	10:17233324-17234833	1510	RP11-124N14.3	antisense	2	FUS,UPF1,
ENSG00000258377	14:49620814-49623480	2667	RP11-649E7.5	antisense	2	FUS,UPF1,
ENSG00000249786	3:15436170-15451602	15,433	EAF1-AS1	antisense	2	eIF4AIII,UPF1,
ENSG00000263424	18:67506588-67514030	7443	CTD-2541J13.2	antisense	2	DGCR8,UPF1,
ENSG00000267257	18:58535414-58538552	3139	RP11-1151B14.4	antisense	2	DGCR8,FUS,
ENSG00000255864	12:24213255-24562590	349,336	RP11-444D3.1	lincRNA	2	eIF4AIII,FUS,
ENSG00000227112	6:128505124-128506276	1153	RP1-86D1.4	antisense	1	FUS,
ENSG00000258908	14:20474788-20477089	2302	RP11-203M5.8	lincRNA	1	UPF1,
ENSG00000263065	16:15741150-15741791	642	AF001548.6	antisense	1	FUS,
ENSG00000249835	5:83541476-83581320	39,845	VCAN-AS1	antisense	1	eIF4AIII,
ENSG00000268309	19:16551772-16552328	557	CTD-3222D19.11	antisense	1	UPF1,
ENSG00000236498	2:61868431-61886082	17,652	AC107081.5	antisense	1	UPF1,
ENSG00000264558	17:47682416-47682683	268	RP11-138C9.1	antisense	1	FUS,
ENSG00000250186	17:49404080-49405197	1118	RP11-1079K10.4	antisense	1	UPF1,
ENSG00000253174	8:41540380-41545044	4665	RP11-360L9.7	antisense	1	UPF1,
ENSG00000254682	11:71448673-71452157	3485	RP11-660L16.2	antisense	1	U2AF65,
ENSG00000234883	21:25561908-25575168	13,261	MIR155HG	lincRNA	1	PTB,
ENSG00000267886	19:23075200-23100361	25,162	CTD-2291D10.4	lincRNA	1	DGCR8,
ENSG00000269292	19:46609276-46610779	1504	CTB-12A17.3	antisense	1	UPF1,
ENSG00000268854	19:50480118-50483351	3234	CTD-2545M3.2	antisense	1	UPF1,
ENSG00000262831	17:81843164-81843958	795	RP11-498C9.2	antisense	1	UPF1,
ENSG00000267512	19:13139616-13141147	1532	CTC-250I14.3	antisense	1	UPF1,
ENSG00000203279	9:97200474-97238700	38,227	RP11-498P14.5	lincRNA	1	eIF4AIII,
ENSG00000232527	1:144227029-144250288	23,260	RP11-14N7.2	lincRNA	1	eIF4AIII,
ENSG00000257181	12:68841287-68843237	1951	RP11-611O2.5	antisense	1	UPF1,
ENSG00000236886	2:216694463-216994079	299,617	AC007563.5	antisense	1	FUS,
ENSG00000259627	15:63070024-63071911	1888	RP11-244F12.2	antisense	1	DGCR8,
ENSG00000260349	16:9105833-9107174	1342	RP11-473I1.5	antisense	1	UPF1,
ENSG00000265784	17:38918800-38921769	2970	RP1-56K13.3	antisense	1	UPF1,
ENSG00000261295	X:100673329-100673981	653	RP11-524D16__A.3	antisense	1	eIF4AIII,
ENSG00000236581	13:33271378-33281334	9957	STARD13-AS	processed_transcript	1	eIF4AIII,
ENSG00000279753	19:1038726-1039064	339	AC011558.5	TEC	1	UPF1,
ENSG00000259498	15:63046033-63049387	3355	RP11-244F12.3	antisense	1	FUS,
ENSG00000227248	13:107788341-107835451	47,111	FAM155A-IT1	sense_intronic	1	FUS,

**Table 2:** RNA and protein expression of RNA binding proteins of lncRNAs identified in this study.

**RBP**	**Number of lncRNA interacted with**	**The list of lncRNAs that interact with RBP**	**RNA seq Reads (FPKM)**					**Fold change at mRNA level**		**Fold-changes of RBP at protein level**	
			**Mock**	**Ad2-6 hpi**	**Ad2-12 hpi**	**Ad2-24 hpi**	**Ad2-36 hpi**	**Ad2-24 hpi/M**	**Ad2-36 hpi/M**	**Ad2-24 hpi/M**	**Ad2-36 hpi/M**
eIF4AIII	112	NEAT1,DLEU2,SNHG10,SNHG1,SNHG5,SNHG6,SNHG12,XIST,SNHG7,MIR17HG,SNHG9,CTD-2033A16.1,RP11-552M11.4,BCYRN1,CTA-363E6.7,ENTPD1-AS1,RP11-324I22.4,EAF1-AS1,RP11-524D16__A.3,AC012146.7,RP11-14N7.2,CTD-2349P21.9,AC007246.3,ERVK3-1,RP11-442H21.2,SNHG3,RP11-727A23.5,RP11-166D19.1,AC002117.1,AC017002.2,RP11-603J24.17,RP11-629O1.2,RP11-295G20.2,RP11-444D3.1,HNRNPU-AS1,AC009133.15,RP11-783K16.5,SNORA71A,RP11-111M22.3,CTA-29F11.1,RP11-29G8.3,TMEM161B-AS1,CTD-2231E14.8,HAS2-AS1,RP11-303E16.2,RP11-820L6.1,SNHG8,CTD-2245F17.3,LINC00641,RP11-473M20.16,SNHG15,MIR22HG,FTX,HOXA-AS2,SNHG14,AC132217.4,LINC00152,AC093323.3,STARD13-AS,MFI2-AS1,CRNDE,CTC-459F4.3,LINC00657,MEG3,RP11-6O2.4,RP11-206L10.9,VCAN-AS1,ZFAS1,MMP24-AS1,THAP9-AS1,RP11-77P6.2,RP5-940J5.9,CTD-2651B20.6,RP11-159D12.2,RP11-316M1.12,ANKRD10-IT1,CDKN2B-AS1,CTD-3099C6.9,RP11-1094M14.11,RPPH1,RP11-572O17.1,CTD-3131K8.2,RP5-875O13.1,RP11-498P14.5,DANCR,FENDRR,RP11-355O1.11,AC009133.12,RP11-265N6.2,LINC00630,CTB-31O20.2,BDNF-AS,CTD-3184A7.4,SCARNA10,SCARNA9,AC159540.1,SCARNA2,HOTAIRM1,ZNF674-AS1,OIP5-AS1,SNHG16,LINC00475,RP11-658F2.8,RP13-131K19.2,AC137934.1,RNU4ATAC,RNU11,MAPKAPK5-AS1,PCBP1-AS1,COX10-AS1,AC005154.6,CTD-2006C1.2,	21.5	24.3	29.2	49.7	34.6	2.3	1.6	1,3^a^	1.6
UPF1	104	SNHG5,NEAT1,SNHG9,SNHG1,MIR17HG,SNHG6,SNHG10,SNHG7,SNHG12,DLEU2,XIST,CTD-3222D19.11,CTD-2033A16.1,RP11-552M11.4,RP11-498C9.2,BCYRN1,CTB-12A17.3,ENTPD1-AS1,RP11-324I22.4,EAF1-AS1,CTD-2349P21.9,RP11-611O2.5,AC007246.3,ERVK3-1,RP11-442H21.2,SNHG3,RP11-473I1.5,RP11-166D19.1,AC002117.1,RP11-804A23.4,RP11-603J24.17,RP11-629O1.2,RP11-295G20.2,RNU6-2,HNRNPU-AS1,RP11-783K16.5,CTD-2545M3.2,SNORA71A,RP11-111M22.3,AC011558.5,RP11-29G8.3,RP11-124N14.3,TMEM161B-AS1,CTD-2231E14.8,HAS2-AS1,RP11-303E16.2,RP11-820L6.1,SNHG8,RP11-1079K10.4,LINC00641,RP11-473M20.16,SNHG15,MIR22HG,FTX,HOXA-AS2,SNHG14,AC093323.3,LINC00152,RP1-56K13.3,CTC-425F1.4,CRNDE,RP11-108M9.3,CTC-459F4.3,LINC00657,RP11-203M5.8,RP11-649E7.5,AC107081.5,ZFAS1,MMP24-AS1,THAP9-AS1,CTD-2541J13.2,RP11-867G23.4,RP5-940J5.9,RP11-360L9.7,CTD-2651B20.6,RP11-316M1.12,CDKN2B-AS1,CTD-3099C6.9,CTC-250I14.3,RP11-1094M14.11,RPPH1,DANCR,FENDRR,RP11-355O1.11,RP11-265N6.2,LINC00630,BDNF-AS,CTD-3184A7.4,SCARNA10,SCARNA9,SCARNA2,HOTAIRM1,ZNF674-AS1,OIP5-AS1,SNHG16,RP11-658F2.8,RP13-131K19.2,RNU4ATAC,AC005523.2,RNU11,MAPKAPK5-AS1,PCBP1-AS1,COX10-AS1,AC005154.6,	11.6	11.8	12.9	24.5	24.1	2.1	2.1	1.5	1.9
FUS	96	SNHG12,DLEU2,SNHG1,MIR17HG,SNHG6,SNHG10,SNHG9,NEAT1,SNHG7,SNHG5,MEG8,XIST,RP11-552M11.4,BCYRN1,CTA-363E6.7,AC007563.5,RP11-138C9.1,ENTPD1-AS1,RP11-324I22.4,CTD-2349P21.9,AC007246.3,ERVK3-1,SNHG3,RP11-166D19.1,RP1-86D1.4,AC002117.1,RP11-804A23.4,AC017002.2,RP11-444D3.1,HNRNPU-AS1,CTA-29F11.1,RP11-29G8.3,RP11-124N14.3,TMEM161B-AS1,CTD-2231E14.8,HAS2-AS1,SNHG8,CTD-2245F17.3,LINC00641,RP11-244F12.3,SNHG15,MIR22HG,FTX,AF001548.6,HOXA-AS2,SNHG14,AC132217.4,AC093323.3,LINC00152,CTC-425F1.4,MIR143HG,CRNDE,CTC-459F4.3,LINC00657,MEG3,RP11-6O2.4,RP11-649E7.5,FAM155A-IT1,RP11-206L10.9,ZFAS1,MMP24-AS1,THAP9-AS1,RP11-77P6.2,RP11-867G23.4,RP11-1151B14.4,RP11-159D12.2,ANKRD10-IT1,CDKN2B-AS1,RP11-1094M14.11,RPPH1,RP11-572O17.1,CTD-3131K8.2,DANCR,FENDRR,LINC00630,CTB-31O20.2,BDNF-AS,AC006116.24,CTD-3184A7.4,SCARNA10,SCARNA9,AC159540.1,SCARNA2,HOTAIRM1,OIP5-AS1,SNHG16,LINC00475,RP11-658F2.8,RP13-131K19.2,AC137934.1,RNU4ATAC,AC005523.2,RNU11,MAPKAPK5-AS1,PCBP1-AS1,AC005154.6,	5.3	3.8	5.7	8.4	9.3	1.6	1.8	1.5	1.5
U2AF65	72	SNHG10,SNHG9,DLEU2,SNHG12,SNHG1,SNHG7,SNHG6,MIR17HG,NEAT1,SNHG5,RP11-552M11.4,BCYRN1,ENTPD1-AS1,AC012146.7,AC007246.3,ERVK3-1,SNHG3,RP11-727A23.5,RP11-166D19.1,AC002117.1,RNU6-2,HNRNPU-AS1,RP11-111M22.3,CTA-29F11.1,RP11-29G8.3,TMEM161B-AS1,RP11-303E16.2,RP11-820L6.1,SNHG8,RP11-660L16.2,LINC00641,SNHG15,MIR22HG,FTX,HOXA-AS2,AC093323.3,LINC00152,MFI2-AS1,CRNDE,RP11-108M9.3,CTC-459F4.3,LINC00657,RP11-206L10.9,ZFAS1,MMP24-AS1,THAP9-AS1,RP11-159D12.2,ANKRD10-IT1,CDKN2B-AS1,RP11-1094M14.11,RPPH1,RP11-572O17.1,RP5-875O13.1,DANCR,RP11-355O1.11,AC009133.12,LINC00630,CTB-31O20.2,CTD-3184A7.4,SCARNA10,SCARNA2,HOTAIRM1,ZNF674-AS1,OIP5-AS1,SNHG16,RP11-658F2.8,RNU4ATAC,RNU11,MAPKAPK5-AS1,PCBP1-AS1,COX10-AS1,AC005154.6,	–	–	–	–	–	–	–	–	–
DGCR8	64	SNHG12,SNHG1,MIR17HG,NEAT1,SNHG9,DLEU2,SNHG7,XIST,SNHG5,SNHG6,RN7SL1,AC012146.7,AC007246.3,ERVK3-1,RP11-442H21.2,SNHG3,RP11-727A23.5,RP11-166D19.1,RP11-804A23.4,HNRNPU-AS1,SNORA71A,RP11-29G8.3,TMEM161B-AS1,RP11-303E16.2,SNHG8,LINC00641,RP11-473M20.16,SNHG15,MIR22HG,FTX,HOXA-AS2,SNHG14,AC093323.3,RP11-244F12.2,CRNDE,RP11-108M9.3,CTC-459F4.3,LINC00657,MEG3,MMP24-AS1,THAP9-AS1,CTD-2541J13.2,RP11-1151B14.4,CTD-2651B20.6,RP11-159D12.2,CDKN2B-AS1,RPPH1,RP5-875O13.1,DANCR,FENDRR,LINC00630,CTB-31O20.2,CTD-3184A7.4,SCARNA10,SCARNA9,SCARNA2,HOTAIRM1,OIP5-AS1,SNHG16,CTD-2291D10.4,RNU4ATAC,RNU11,PCBP1-AS1,AC005154.6,	9.4	11.1	10.5	8.9	2.7	−1.1	−3.5	–	–
hnRNPC	50	SNHG1,SNHG5,DLEU2,SNHG6,SNHG7,SNHG10,NEAT1,MIR17HG,SNHG12,RP11-552M11.4,ENTPD1-AS1,RN7SL1,SNHG3,RP11-727A23.5,RP11-166D19.1,RNU6-2,HNRNPU-AS1,RP11-29G8.3,TMEM161B-AS1,RP11-820L6.1,SNHG8,LINC00641,SNHG15,FTX,AC093323.3,MIR143HG,CRNDE,RP11-108M9.3,CTC-459F4.3,LINC00657,THAP9-AS1,CTD-2651B20.6,RP11-159D12.2,ANKRD10-IT1,FENDRR,RP11-355O1.11,LINC00630,CTB-31O20.2,SCARNA10,SCARNA9,SCARNA2,HOTAIRM1,ZNF674-AS1,OIP5-AS1,SNHG16,RNU4ATAC,RNU11,PCBP1-AS1,COX10-AS1,AC005154.6,	157.8	169.3	181.6	289.6	474.8	1.8	3	1.1	1.1
FMRP	49	NEAT1,DLEU2,SNHG9,SNHG12,SNHG5,SNHG7,MIR17HG,SNHG1,XIST,SNHG6,SNHG10,RP11-552M11.4,ENTPD1-AS1,RN7SL1,ERVK3-1,SNHG3,HNRNPU-AS1,SNORA71A,RP11-29G8.3,SNHG8,LINC00641,RP11-473M20.16,SNHG15,MIR22HG,HOXA-AS2,AC093323.3,CRNDE,CTC-459F4.3,LINC00657,ZFAS1,MMP24-AS1,CTD-2651B20.6,CTD-3099C6.9,RP11-1094M14.11,RPPH1,RP5-875O13.1,DANCR,LINC00630,CTB-31O20.2,SCARNA10,SCARNA9,SCARNA2,ZNF674-AS1,OIP5-AS1,SNHG16,RNU4ATAC,RNU11,MAPKAPK5-AS1,PCBP1-AS1,	–	–	–	–	–	–	–	–b	–
TIAL1	48	SNHG1,DLEU2,MIR17HG,SNHG12,SNHG5,SNHG6,NEAT1,XIST,RP11-552M11.4,BCYRN1,ENTPD1-AS1,AC012146.7,AC007246.3,ERVK3-1,SNHG3,RP11-727A23.5,AC017002.2,HNRNPU-AS1,RP11-111M22.3,TMEM161B-AS1,RP11-820L6.1,LINC00641,RP11-473M20.16,SNHG15,FTX,SNHG14,AC093323.3,LINC00152,MIR143HG,CRNDE,RP11-108M9.3,CTC-459F4.3,LINC00657,THAP9-AS1,CTD-2651B20.6,RPPH1,DANCR,LINC00630,SCARNA10,SCARNA9,SCARNA2,HOTAIRM1,OIP5-AS1,SNHG16,RNU11,PCBP1-AS1,COX10-AS1,AC005154.6,	14.8	8.7	9.1	6.3	5.5	−2.4	−2.7	1.1	−1.1
PTB	47	SNHG1,MIR17HG,NEAT1,SNHG5,MIR155HG,HNRNPU-AS1,RPPH1,RP11-552M11.4,DANCR,CRNDE,HOXB-AS3,RP11-108M9.3,ENTPD1-AS1,CTC-459F4.3,FENDRR,LINC00657,RP11-355O1.11,RP11-29G8.3,MEG3,SCARNA10,SCARNA9,TMEM161B-AS1,SCARNA2,HOTAIRM1,HAS2-AS1,RP11-303E16.2,ZNF674-AS1,OIP5-AS1,SNHG16,LINC00475,AC007246.3,LINC00641,ERVK3-1,SNHG3,MIR22HG,RNU11,CTD-2651B20.6,PCBP1-AS1,SNHG14,RP11-159D12.2,COX10-AS1,LINC00152,AC093323.3,RP11-629O1.2,AC005154.6,CDKN2B-AS1,CTD-3099C6.9,	–	–	–	–	–	–	–	–	–
LIN28B	39	SNHG10,SNHG1,DLEU2,MIR17HG,SNHG6,SNHG12,NEAT1,XIST,SNHG7,SNHG5,RP11-552M11.4,HOXB-AS3,ENTPD1-AS1,RN7SL1,AC012146.7,ERVK3-1,SNHG3,HNRNPU-AS1,AC009133.15,LINC00641,RP11-473M20.16,MIR22HG,CRNDE,LINC00657,THAP9-AS1,CTD-2651B20.6,CTD-3099C6.9,RP11-1094M14.11,RPPH1,DANCR,LINC00630,SCARNA10,SCARNA2,ZNF674-AS1,OIP5-AS1,SNHG16,RNU11,PCBP1-AS1,AC005154.6,	–	–	–	–	–	–	–	–	–
LIN28A	38	MIR17HG,SNHG12,SNHG6,XIST,SNHG1,NEAT1,SNHG5,SNHG7,RP11-552M11.4,HOXB-AS3,RN7SL1,ERVK3-1,SNHG3,HNRNPU-AS1,SNORA71A,SNHG8,RP11-473M20.16,SNHG15,MIR22HG,CRNDE,RP11-108M9.3,CTC-459F4.3,LINC00657,ZFAS1,MMP24-AS1,CTD-2651B20.6,CDKN2B-AS1,RP11-1094M14.11,RPPH1,SCARNA10,SCARNA2,HOTAIRM1,OIP5-AS1,SNHG16,RNU4ATAC,RNU11,MAPKAPK5-AS1,PCBP1-AS1,	–	–	–	–	–	–	–	–	–
IGF2BP1	34	DLEU2,SNHG1,NEAT1,SNHG6,SNHG7,SNHG5,XIST,HNRNPU-AS1,RP11-1094M14.11,RP11-552M11.4,DANCR,CRNDE,CTC-459F4.3,LINC00657,RP11-29G8.3,CTB-31O20.2,AC012146.7,HOTAIRM1,ZFAS1,RP11-303E16.2,OIP5-AS1,MMP24-AS1,SNHG16,THAP9-AS1,LINC00641,RP11-658F2.8,SNHG3,FTX,MAPKAPK5-AS1,RP11-159D12.2,AC093323.3,RP11-295G20.2,CTD-2006C1.2,ANKRD10-IT1,	8.3	8.2	7.9	13.5	12.7	1.6	1.5	1.1	1.4
IGF2BP2	33	SNHG7,DLEU2,SNHG1,SNHG6,NEAT1,SNHG5,XIST,HNRNPU-AS1,RP11-1094M14.11,RP11-552M11.4,DANCR,CRNDE,CTC-459F4.3,LINC00657,RP11-29G8.3,LINC00630,AC012146.7,ZFAS1,RP11-303E16.2,OIP5-AS1,MMP24-AS1,SNHG16,LINC00641,RP11-658F2.8,SNHG3,FTX,CTD-2651B20.6,RP11-159D12.2,AC093323.3,RP11-295G20.2,CTD-2006C1.2,ANKRD10-IT1,CTD-3099C6.9,	19.9	16.9	8.3	9.5	9.8	−2.1	−2	−1.2	−1.1
IGF2BP3	31	SNHG1,DLEU2,SNHG7,NEAT1,SNHG6,XIST,SNHG5,HNRNPU-AS1,RP11-1094M14.11,RP11-552M11.4,DANCR,CRNDE,CTC-459F4.3,LINC00657,LINC00630,AC012146.7,TMEM161B-AS1,ZFAS1,RP11-303E16.2,OIP5-AS1,MMP24-AS1,SNHG16,LINC00641,RP11-658F2.8,SNHG3,FTX,MAPKAPK5-AS1,RP11-159D12.2,AC093323.3,RP11-295G20.2,ANKRD10-IT1,	28.7	25.7	28.1	17.1	16	−1.7	−1.8	1.1	1.2
ZC3H7B	31	SNHG6,SNHG1,DLEU2,NEAT1,SNHG7,SNHG10,SNHG5,XIST,MIR17HG,RP11-552M11.4,SNHG3,HNRNPU-AS1,SNHG8,LINC00641,SNHG15,MIR22HG,FTX,AC093323.3,CRNDE,LINC00657,ZFAS1,MMP24-AS1,RP11-159D12.2,RP11-1094M14.11,RPPH1,SCARNA10,SCARNA9,OIP5-AS1,SNHG16,RP11-658F2.8,RNU11,	9.7	13.7	10.5	9.1	7.8	−1.1	−1.2	–	–
TDP43	30	SNHG12,NEAT1,SNHG1,SNHG6,SNHG5,MEG8,SNHG7,XIST,RP11-552M11.4,BCYRN1,RN7SL1,AC007246.3,SNHG3,SNHG8,LINC00641,SNHG15,FTX,HOXA-AS2,SNHG14,LINC00657,MEG3,CTD-2651B20.6,RPPH1,SCARNA10,SCARNA9,SCARNA2,OIP5-AS1,SNHG16,RNU11,PCBP1-AS1,	–	–	–	–	–	–	–	–	–
HuR	30	SNHG1,SNHG6,DLEU2,SNHG9,MIR17HG,SNHG7,SNHG10,SNHG12,SNHG5,XIST,RP11-303E16.2,HNRNPU-AS1,ZNF674-AS1,OIP5-AS1,MMP24-AS1,SNHG8,RPPH1,RP11-552M11.4,DANCR,CRNDE,SNHG3,SNHG15,MIR22HG,ENTPD1-AS1,LINC00657,RP11-29G8.3,RP11-159D12.2,AC093323.3,RP11-6O2.4,ZFAS1,	–	–	–	–	–	–	–	–	–
TIA1	29	MIR17HG,SNHG1,DLEU2,NEAT1,SNHG6,XIST,RP11-552M11.4,ENTPD1-AS1,AC007246.3,SNHG3,HNRNPU-AS1,RP11-820L6.1,SNHG8,RP11-473M20.16,SNHG15,FTX,AC093323.3,LINC00152,CRNDE,RP11-108M9.3,LINC00657,MMP24-AS1,THAP9-AS1,CTD-2651B20.6,DANCR,OIP5-AS1,SNHG16,RNU11,PCBP1-AS1,	45.1	31	40.2	34	23.9	−1.3	−1.9	–	–
EWSR1	25	SNHG12,MIR17HG,SNHG1,DLEU2,NEAT1,SNHG7,SNHG5,XIST,ENTPD1-AS1,AC012146.7,SNHG3,HNRNPU-AS1,SNHG8,LINC00641,RP11-473M20.16,SNHG15,CRNDE,LINC00657,AC006116.24,SCARNA10,SCARNA9,OIP5-AS1,SNHG16,RNU11,AC005154.6,	133.3	143.5	118.2	69.6	94.2	−1.9	−1.4	−1.3	−1.5
SFRS1	25	MIR17HG,SNHG1,SNHG12,NEAT1,SNHG7,SNHG5,XIST,AC012146.7,SNHG3,HNRNPU-AS1,SNORA71A,RP11-29G8.3,TMEM161B-AS1,SNHG8,CTD-2245F17.3,SNHG15,FTX,LINC00657,RP11-206L10.9,ZFAS1,CTD-2651B20.6,HOTAIRM1,OIP5-AS1,SNHG16,PCBP1-AS1,	–	–	–	–	–	–	–	–	–
LIN28	23	SNHG12,SNHG6,XIST,SNHG1,SNHG5,SNHG7,RP11-552M11.4,SNHG3,HNRNPU-AS1,RP11-111M22.3,SNHG8,SNHG15,SNHG14,CRNDE,ZFAS1,THAP9-AS1,CTD-2651B20.6,RP5-875O13.1,DANCR,SCARNA10,OIP5-AS1,SNHG16,PCBP1-AS1,	–	–	–	–	–	–	–	–	–
TAF15	22	NEAT1,SNHG1,SNHG10,DLEU2,SNHG7,XIST,AC012146.7,SNHG3,HNRNPU-AS1,SNHG8,SNHG15,CRNDE,CTC-459F4.3,LINC00657,ZFAS1,THAP9-AS1,RP11-159D12.2,RP11-1094M14.11,RPPH1,DANCR,OIP5-AS1,SNHG16,	10.9	10.6	7.6	4.8	2	−2.3	−5.5	−1.2	
FXR2	21	SNHG1,SNHG12,SNHG5,XIST,RN7SL1,SNHG3,HNRNPU-AS1,RP11-29G8.3,LINC00641,RP11-473M20.16,SNHG15,CRNDE,LINC00657,RPPH1,SCARNA10,SCARNA9,SCARNA2,OIP5-AS1,SNHG16,RNU11,PCBP1-AS1,	22.7	25.2	31.7	16.3	7.1	−1.4	−3.2	1.3	1.4
C22ORF28	20	NEAT1,SNHG12,SNHG1,SNHG7,XIST,SNHG5,SNHG6,SNHG9,SNHG3,HNRNPU-AS1,RP11-29G8.3,SNHG8,SNHG15,CTC-459F4.3,LINC00657,ZFAS1,RPPH1,SCARNA10,SNHG16,RNU11,	–	–	–	–	–	–	–	–	–
FUS-mutant	19	DLEU2,SNHG1,NEAT1,SNHG7,XIST,RP11-552M11.4,AC012146.7,SNHG3,HNRNPU-AS1,LINC00641,CRNDE,LINC00657,ZFAS1,THAP9-AS1,RP11-1094M14.11,DANCR,SCARNA9,OIP5-AS1,SNHG16,	–	–	–	–	–	–	–	–	–
CAPRIN1	17	SNHG9,SNHG1,SNHG6,SNHG5,XIST,HNRNPU-AS1,SNHG8,LINC00641,SNHG15,AC093323.3,LINC00657,ZFAS1,THAP9-AS1,RPPH1,DANCR,HOTAIRM1,OIP5-AS1,	84.2	70.2	78.7	79.1	80.2	−1.1	−1.1	1.4	−1.7
MOV10	15	SNHG1,MIR17HG,SNHG6,XIST,RN7SL1,RNU6-2,HNRNPU-AS1,SNHG8,AC093323.3,LINC00657,ZFAS1,SCARNA2,OIP5-AS1,SNHG16,RNU11,	3.4	4.1	4.8	3.9	4.9	1.1	1.4	1.1	1.1
TNRC6	12	NEAT1,SNHG5,SNHG6,SNHG1,XIST,HNRNPU-AS1,RPPH1,CRNDE,LINC00657,RP11-29G8.3,RP11-303E16.2,OIP5-AS1,	–	–	–	–	–	–	–	–	–
C17ORF85	12	SNHG6,SNHG12,SNHG5,SNHG1,XIST,LINC00657,RPPH1,SCARNA10,SCARNA9,SCARNA2,SNHG16,RNU11,	19.6	15.9	15.3	12.2	9.8	−1.6	−2	–	–
PUM2	8	SNHG6,SNHG5,DLEU2,XIST,HNRNPU-AS1,LINC00657,OIP5-AS1,THAP9-AS1,	26	17.4	29.9	37.8	15.3	1.5	−1.7	–	–
ALKBH5	7	SNHG1,NEAT1,XIST,SNHG7,HNRNPU-AS1,RPPH1,RNU11,	14.7	17.1	24.9	32.9	29.8	2.2	2	–	–
QKI	7	SNHG1,NEAT1,SNHG5,HNRNPU-AS1,CTC-459F4.3,LINC00657,SNHG16,	8.8	9.2	6	5.4	4.6	−1.6	−1.9	−1.1	–b
FXR1	5	MIR17HG,XIST,HNRNPU-AS1,LINC00657,OIP5-AS1,	20.7	19.5	20.3	26.1	25.5	1.3	1.2	1.2	1.3

a Fold change in lncRNA, mRNA or protein expression between adenovirus infected and uninfected cells (mock).

b Expression was not detected or low sequencing reads (<10 FPKM).
